# Influence of haem environment on the catalytic properties of the tetrathionate reductase TsdA from *Campylobacter jejuni*

**DOI:** 10.1042/BSR20160457

**Published:** 2016-12-09

**Authors:** Julia M. Kurth, Julea N. Butt, David J. Kelly, Christiane Dahl

**Affiliations:** * Institut für Mikrobiologie & Biotechnologie, Rheinische Friedrich-Wilhelms-Universität Bonn, D-53115 Bonn, Germany; † Centre for Molecular and Structural Biochemistry, School of Chemistry, and School of Biological Sciences, University of East Anglia, Norwich Research Park, Norwich NR4 7TJ, U.K.; ‡ Department of Molecular Biology and Biotechnology, The University of Sheffield, Firth Court, Western Bank, Sheffield S10 2TN, U.K.

**Keywords:** axial haem ligation, cytochrome *c*, reaction directionality, tetrathionate reductase, thiosulfate

## Abstract

Bifunctional dihaem cytochrome *c* thiosulfate dehydrogenases/tetrathionate reductases (TsdA) exhibit different catalytic properties depending on the source organism. In the human food-borne intestinal pathogen *Campylobacter jejuni*, TsdA functions as a tetrathionate reductase enabling respiration with tetrathionate as an alternative electron acceptor. In the present study, evidence is provided that Cys^138^ and Met^255^ serve as the sixth ligands of Haem 1 and Haem 2 respectively, in the oxidized *Cj*TsdA wt protein. Replacement of Cys^138^ resulted in a virtually inactive enzyme, confirming Haem 1 as the active site haem. Significantly, TsdA variants carrying amino acid exchanges in the vicinity of the electron-transferring Haem 2 (Met^255^, Asn^254^ and Lys^252^) exhibited markedly altered catalytic properties of the enzyme, showing these residues play a key role in the physiological function of TsdA. The growth phenotypes and tetrathionate reductase activities of a series of ΔtsdA/*tsdA complementation strains constructed in the original host *C. jejuni* 81116, showed that *in vivo*, the TsdA variants exhibited the same catalytic properties as the pure, recombinantly produced enzymes. However, variants that catalysed tetrathionate reduction more effectively than the wild-type enzyme did not allow better growth.

## Abstract (precis)

In the present study, we provide a detailed analysis of the catalytic properties of the bifunctional thiosulfate dehydrogenases/tetrathionate reductases (TsdA) of the human food-borne pathogen *Campylobacter jejuni*. Structural differences in the immediate environment of Haem 2 were shown to influence the reaction directionality.

## INTRODUCTION

Although tetrathionate (^−^O_3_S–S–S–SO_3_^−^) has long been known to be used by some bacteria as an electron acceptor under anaerobic conditions, recent evidence suggests that the biochemical and environmental significance of tetrathionate respiration has been hugely underestimated [1,2]. In a single step requiring input of two electrons, tetrathionate is reduced to two molecules of thiosulfate (^−^S–SO_3_^−^). The midpoint reduction potential of the tetrathionate/thiosulfate couple was only very recently determined by experimental means and found to be +198 mV compared with standard hydrogen electrode (SHE) [1], a value considerably more positive than the calculation-based value of +24 mV cited in many bacterial bioenergetics studies [3]. As a consequence, more free energy is available to be harnessed during the respiratory reduction of tetrathionate than was previously recognized. Furthermore, the high relevance of tetrathionate as an *in vivo* electron acceptor for bacterial pathogenesis is emphasized by the finding that the human intestinal pathogen *Salmonella typhimurium* induces host-driven formation of tetrathionate from thiosulfate by reactive oxygen species produced during inflammation [4]. The tetrathionate thus formed is used as terminal electron acceptor providing *S. typhimurium* with a growth advantage over the majority of the commensal flora lacking the capacity for tetrathionate reduction. Nevertheless, the extent to which this capacity is exploited by other enteric pathogens has yet to be fully appreciated. For example, although it has long been known that *Citrobacter* and *Proteus* species are also able to perform tetrathionate respiration [5], the mechanism and *in vivo* significance of this have not been studied.

In terms of the burden of disease, the microaerophilic food-borne pathogen *Campylobacter jejuni* is responsible for the majority of cases of bacterial gastroenteritis in the Western world, often being far more prevalent than any other enteric bacterium including *Salmonella* [6]. Normally commensal in the intestinal tract of chickens, in humans *C. jejuni* causes acute bloody diarrhoea and in some cases the sequelae include neuromuscular paralysis and even death [7,8]. Previously, it was found that some strains of *C. jejuni* are capable of tetrathionate respiration [2] and it was suggested that this plays an important role in growth in the oxygen-limited human intestinal mucosa; the ability to respire tetrathionate is thus relevant for understanding the pathogenicity of the organism [2]. The finding that *C. jejuni* strains are capable of tetrathionate reduction and that this can stimulate growth under oxygen-limited conditions added a further dimension to the complex, branched electron transport pathways employed in this organism. Other alternatives to oxygen as the terminal respiratory electron acceptor include fumarate, nitrite, trimethylamine *N*-oxide and dimethyl sulfoxide [9–11].

**Figure 1:**
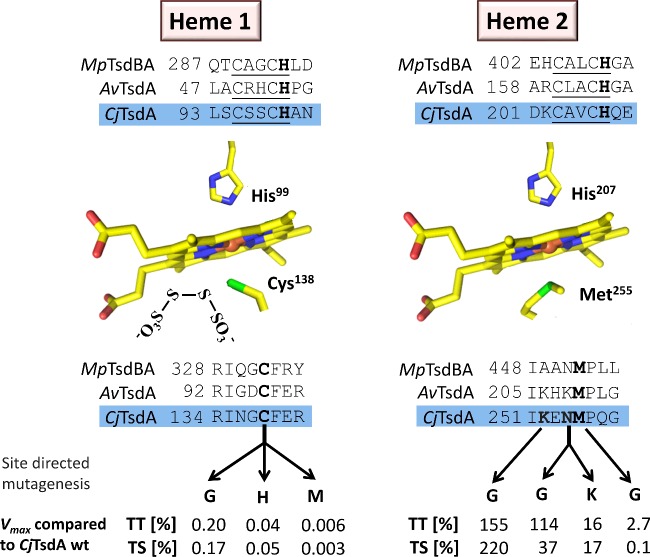
Schematic overview of Haem 1 and Haem 2 environments in Tsd(B)A proteins Relevant sequence alignments are shown for TsdA from *C. jejuni* (*Cj*TsdA), *A. vinosum* (*Av*TsdA) and the TsdBA fusion protein from *M. purpuratum* (*Mp*TsdBA). Amino acid numbers are given for the recombinant proteins without signal peptides. In case of *Cj*TsdA, the sequence of the N-terminal Strep-tag is included. In the central left panel, a tetrathionate molecule is shown in vicinity of the active site Haem 1 iron-ligating cysteine, based on the *Av*TsdA crystal structure [16]. Amino acid numbers in the central panels refer to C*j*TsdA. In the lower part of the figure, changes in the environments of Haem 1 and Haem 2 are indicated that were introduced by site-directed mutagenesis. The effects of these changes on maximal reaction velocity are listed as percent of *V*_max_ for the wild-type enzyme in the tetrathionate-reducing (TT) and the thiosulfate-oxidizing (TS) direction.

Surprisingly, the enzyme catalysing tetrathionate reduction in *C. jejuni* was found to be distinct from other known tetrathionate reductases, i.e. the iron–sulfur molybdoenzyme TtrABC found in *S. typhimurium* [12,13] and the octahaem Otr enzyme from *Shewanella oneidensis* [14]. Instead, the *Campylobacter* enzyme belongs to a novel, widely-distributed class of bifunctional thiosulfate dehydrogenase/tetrathionate reductases (TsdA) residing in the bacterial periplasm. TsdA enzymes represent a distinct type of dihaem *c-*type cytochrome [2,15,16]. An axial histidine/cysteine ligation of the central iron atom has been established for the active site haem (Haem 1) of the enzyme from *Allochromatium vinosum*, *Av*TsdA [15,16] (Figure 1). This type of ligation is rare among prokaryotes and appears to be of special importance in sulfur-based energy metabolism. In *Av*TsdA, Haem 2 exhibits axial His/Lys and His/Met co-ordination in the oxidized and reduced state respectively [16].

Although all TsdA enzymes characterized to date catalyse both the reduction of tetrathionate and the oxidation of two molecules of thiosulfate to tetrathionate at measurable rates, the enzymes from different bacteria still exhibit very different catalytic properties *in vitro* and have been shown to possess differences in reaction directionality using biochemical assays with redox dyes and also electrochemical protein film voltammetry [1,2,15,16]. The extent to which tetrathionate production or thiosulfate oxidation is favoured, is intrinsic to each TsdA. The *A. vinosum* enzyme catalyses tetrathionate reduction at a very low rate that is only measurable in colorimetric solution assays [15,16]. No evidence for reductive catalysis by *Av*TsdA could be found via protein film cyclic voltammetry whereas *Cj*TsdA appeared clearly biased towards tetrathionate reduction relative to thiosulfate oxidation in these experiments [1]. On graphite electrodes, the enzyme from the anoxygenic phototrophic sulfur bacterium *Marichromatium purpuratum*, *Mp*TsdBA, displays higher catalytic rates for thiosulfate oxidation than tetrathionate reduction revealing this enzyme's bias to oxidative catalysis [1]. The enzymatic features measured *in vitro* are in line with the different physiological functions of the TsdA enzymes which can enable either the use of tetrathionate as a terminal electron acceptor as in *C. jejuni* or the use of thiosulfate as an electron donor for respiration or photosynthesis as is the case in *A. vinosum* or *M. purpuratum*.

Very many if not all sequenced *C. jejuni* strains harbour a second *tsdA*-related gene. The corresponding protein CccC from *C. jejuni* strain NCTC 11168 (formerly Cj0037; C8j_0040 in strain 81116) has recently been identified as an efficient electron donor to the *cb*-oxidase [17]. These proteins contain a methionine in place of the probable Haem 1 ligating cysteine in TsdA. In addition, variation is apparent in the vicinity of the probable Haem 2-ligating methionine. Although all bona fide *C. jejuni tsdA* sequences encode an asparagine one residue upstream of the methionine, an alanine is present in the C8j_0040-related sequences. In a previous experimental approach towards the *in vivo* role of TsdA and related proteins in *C. jejuni* 81116, gene inactivation and complementation showed that the protein corresponding to typical TsdA is absolutely essential for growth on tetrathionate under oxygen limitation. A *tsdA* null mutant still had low tetrathionate reductase and thiosulfate dehydrogenase activities and slowly reduced tetrathionate probably due to low activity of CccC (Cj8_0040) as tetrathionate reductase, albeit this did not support growth [2].

There are still significant gaps in our knowledge about the physiological roles and biochemical properties of the proteins of the TsdA family. The enzyme from the sulfur oxidizer *A. vinosum* is the only one for which variants have so far been studied structurally and by UV–Vis spectroscopy. Kinetic characterization was initiated and showed that the replacement of either one of the Haem 2 distal axial ligands lysine or methionine did not render the enzyme catalytically inactive *in vitro* [15,16]. Initial characterization by UV–Vis absorption spectroscopy indicated that TsdA from *C. jejuni* 81116 has similar properties as the protein from *A. vinosum* [2]. However, experimental evidence has so far not been available providing conclusive information about axial haem iron ligation in the *C. jejuni* enzyme. It has therefore not been possible to assess whether structural differences in the immediate environment of one or both haems may be determinants underlying the enzyme's adaptation to catalysis in the tetrathionate-reducing direction [1]. Detailed kinetic data for a TsdA enzyme adapted to tetrathionate reduction is neither available for a wild-type enzyme nor have any variants been produced or analysed. *In vivo* data for performance of enzyme variants in the original host organism are so far not available for any TsdA. In the present study we address these areas, initially by characterization of pure recombinant *Cj*TsdA variants, which have allowed us to assess whether the nature of the haem-ligating residues alters the catalytic properties of the enzyme and whether both or only one catalytic direction(s) are affected. In addition, we have determined by *in vivo* complementation of the *C. jejuni* 81116 *tsdA* null mutant with a set of variant TsdAs, the growth capabilities and tetrathionate reduction rates as well as specific tetrathionate reductase and thiosulfate dehydrogenase activities in crude extracts of the resulting strains.

## MATERIALS AND METHODS

### Bacterial strains, plasmids and growth conditions

Table 1 lists the bacterial strains and plasmids used for the present study. *Escherichia coli* BL21 (DE3) was used for recombinant protein production and was grown in LB medium. *E. coli* DH5α was used for molecular cloning. Wild-type and mutant strains of *C. jejuni* 81116 (NCTC 11828) were used for *in vivo* studies.

**Table 1:** Strains and plasmids used in the present study

Strains and plasmids	Description	References or sources
Strains		
*E. coli* DH5α	F^−^ φ80d *lacZ*ΔM15 Δ(*lacZYA-argF*)*U169 recA1 endA1hsdR17* (rk^−^ mk^+^) *supE44 λ^−^thi-1 gyrA relA1*	[37]
*E. coli* BL21 (DE3)	F^−^ *ompT hsdS_B_* (r_B_^−^m_B_^−^) *gal dcm met*(DE3)	Novagen
*C. jejuni* 81116 (NCTC 11828)	Wild-type	[38]
*C. jejuni* 81116 Δ*tsdA*	Kanr; insertion of kanamycin resistance cassette in place of *c8j_0815*	[2]
*C. jejuni* 81116 ΔtsdA/*tsdA wt	Kanr, Cmr; Δ*tsdA* mutant complented with *c8j_0815* in *cj0046* pseudogene locus behind fdxA promotor	The present study
*C. jejuni* 81116 ΔtsdA/*tsdA CG	Kanr, Cmr; Δ*tsdA* mutant complented with *tsdA* C138G in *cj0046* pseudogene locus behind fdxA promotor	The present study
*C. jejuni* 81116 ΔtsdA/*tsdA MG	Kanr, Cmr; Δ*tsdA* mutant complented with *tsdA* M255G in *cj0046* pseudogene locus behind fdxA promotor	The present study
*C. jejuni* 81116 ΔtsdA/*tsdA NG	Kanr, Cmr; Δ*tsdA* mutant complented with *tsdA* N254G in *cj0046* pseudogene locus behind fdxA promotor	The present study
*C. jejuni* 81116 ΔtsdA/*tsdA KG	Kanr, Cmr; Δ*tsdA* mutant complented with *tsdA* K252G in *cj0046* pseudogene locus behind fdxA promotor	The present study
Plasmids		
pEC86	Cmr, product from pEC66 and pACYC184 with *E. coli ccmABCDEFGH* genes	[20]
pC46-fdxA	*C. jejuni* complementation vector for integration at the *cj0046c* pseudogene locus, expressing cloned genes from the *fdxA* promoter	[39]
pET-N-Strep-TsdACj	Apr; N-terminal Strep-tag, f1 origin, T7 Promoter, NcoI-HindIII fragment of PCR amplified *tsdA* in digested pETStrepDsrJ	[1]
pET-N-Strep-TsdACj-C138G	Apr; C138G mutation introduced into pET-N-Strep-TsdACj	The present study
pET-N-Strep-TsdACj-C138H	Apr; C138H mutation introduced into pET-N-Strep-TsdACj	The present study
pET-N-Strep-TsdACj-C138M	Apr; C138M mutation introduced into pET-N-Strep-TsdACj	The present study
pET-N-Strep-TsdACj-M255G	Apr; M255G mutation introduced into pET-N-Strep-TsdACj	The present study
pET-N-Strep-TsdACj-N254G	Apr; N254G mutation introduced into pET-N-Strep-TsdACj	The present study
pET-N-Strep-TsdACj-N254K	Apr; N254K mutation introduced into pET-N-Strep-TsdACj	The present study
pET-N-Strep-TsdACj-K252G	Apr; K252G mutation introduced into pET-N-Strep-TsdACj	The present study
pET-N-Strep-TsdACj-KMG	Apr; K252G mutation introduced into pET-N-Strep-TsdACj-M255G	The present study
pET-N-Strep-TsdACj-NMG	Apr; N254G mutation introduced into pET-N-Strep-TsdACj-M255G	The present study
pET-N-Strep-TsdACj-KNMG	Apr; K252G andN254G mutation introduced into pET-N-Strep-TsdACj-M255G	The present study
pC46-fdxA-0815HIS	Cmr; *c8j_0815* cloned into pC46-fdxA to produce C-terminal His-tagged TsdA in *C. jejuni*	The present study
pC46-fdxA-TsdACj-C138G	Cmr, C138G mutation introduced into pC46-fdxA-0815	The present study
pC46-fdxA-TsdACj-M255G	Cmr, M255G mutation introduced into pC46-fdxA-0815	The present study
pC46-fdxA-TsdACj-N254G	Cmr, N254G mutation introduced into pC46-fdxA-0815	The present study
pC46-fdxA-TsdACj-K252G	Cmr, K252G mutation introduced into pC46-fdxA-0815	The present study

### Recombinant DNA techniques

All general molecular genetics techniques were described earlier [18]. Restriction enzymes, T4 ligase and *Pfu* or *Phusion* DNA polymerase were obtained from Thermo Scientific or NEB and used according to the manufacturer's instructions. Oligonucleotides for cloning were obtained from Eurofins MWG.

### Construction of expression plasmids and site-directed mutagenesis

The *C. jejuni tsdA* gene coding for the mature protein without the signal peptide was amplified and cloned as described earlier [1]. Point mutations were introduced into *tsdA* by overlap extension [19] using standard PCR with *Pfu* DNA polymerase and pET-N-Strep-TsdACj [1] as the template (Table 1). For the TsdA–C138G exchange, two fragments were amplified with the following primers: for the first fragment pET-N-TsdACj_fw and tsdACj-C-Gly_rev, for the second fragment tsdACj-C-Gly_fw and pET-N-TsdACj_rev (Table 2). Both fragments were used as templates for amplification of the complete *tsdA* gene carrying the desired point mutation. In this step, pET-N-TsdACj_fw and pET-N-TsdACj_rev served as primers (Table 2). The resulting fragment was restricted with BamHI and HindIII and cloned into pET-N-Strep-TsdACj resulting in plasmid pET-N-Strep-TsdACj-C138G. The following plasmids were generated applying the same general strategy: pET-N-Strep-TsdACj-C138G, pET-N-Strep-TsdACj-C138H, pET-N-Strep-TsdACj-C138M, pET-N-Strep-TsdACj-M255G, pET-N-Strep-TsdACj-N254G pET-N-Strep-TsdACj-N254K and pET-N-Strep-TsdACj-K252G (Table 1). For construction of pET-N-Strep-TsdACj-KMG, pET-N-Strep-TsdACj-NMG and pET-N-Strep-TsdACj-KNMG pET-N-Strep-TsdACj-M255G was used as template in PCR.

**Table 2:** Primer used in the present study

Primer	Sequence 5′–3′	References
pET-N-TsdACj_fw	CCGGATCCTTAGATCCAAATTTG	[1]
pET-N-TsdACj_rev	CCGAAGCTTCTACTTCTTGATCA	[1]
0815-com-F/ATG	GGAGCGTCTCACATGAATAAATTTT CTATAGTTTTAACTTTG	The present study
0815-com-R5XHis	ACGTCTCACATGTTAATGATGATGATGTTTTTTGATCATATTTGTA	The present study
tsdACj-C-Gly_fw	GATCAATGGCGGTTTTGAACGCT	The present study
tsdACj-C-Gly_rev	AGCGTTCAAAACCGCCATTGATC	The present study
tsdACj-C-His_fw	GATCAATGGCCATTTTGAACGCT	The present study
tsdACj-C-His_rev	AGCGTTCAAAATGGCCATTGATC	The present study
tsdACj-C-Met_fw	GATCAATGGCATGTTTGAACGCT	The present study
tsdACj-C-Met_rev	AGCGTTCAAACATGCCATTGATC	The present study
tsdACj-M-Gly_fw	TAAAGAAAATGGTCCTCAAGGTG	The present study
tsdACj-M-Gly_rev	CACCTTGAGGACCATTTTCTTTA	The present study
tsdACj-N-Gly_fw	TATTAAAGAAGGTATGCCTCAAGG	The present study
tsdACj-N-Gly_rev	CCTTGAGGCATACCTTCTTTAATA	The present study
tsdACj-N-Lys_fw	TATTAAAGAAAAGATGCCTCAAGG	The present study
tsdACj-N-Lys_rev	CCTTGAGGCATCTTTTCTTTAATA	The present study
tsdACj-K-Gly_fw	CTTCTTATATTGGCGAAAATATGC	The present study
tsdACj-K-Gly_rev	GCATATTTTCGCCAATATAAGAAG	The present study
tsdACj-NM-Gly_fw	TATTAAAGAAGGTGGTCCTCAAG	The present study
tsdACj-NM-Gly_rev	CTTGAGGACCACCTTCTTTAATA	The present study
tsdACj-KM-Gly_fw	TTCTTATATTGGCGAAAATGGTCC	The present study
tsdACj-KM-Gly_rev	GGACCATTTTCGCCAATATAAGAA	The present study
tsdACj-KNM-Gly_fw	TTATATTGGAGAAGGTGGTCCTC	The present study
tsdACj-KNM-Gly_fw	GAGGACCACCTTCTCCAATATAA	The present study
pC46_TsdA_Mfe_fw	GCCCAATTGAAAATCTAAGTAAAAT	The present study
pC46_TsdA_Cla_rev	TGAATCGATAAGGGAATATAGTATT	The present study
p46-Scr-F	CCACTCCTGAAGATGGAACAC	The present study
p46-Scr-R	ATCATTACAAGAGCTGAAAACCATAC	The present study
Cat-Scr-F	AAAAGTTATACCCAACTCTTTTATATGGAG	The present study
Cj46-Scr-R	GTGCAAGTTTTAGATCAGTTTATGGA	The present study

### Overproduction, purification and preparation of recombinant TsdA wild-type and variant proteins

Single colonies of *E. coli* BL21(DE3) containing pET-N-Strep-TsdACj or one of the *tsdA* variant expression plasmids in addition to plasmid pEC86 [20] were inoculated in 800 ml batches of LB medium containing 100 μg·ml^−1^ ampicillin and 25 μg·ml^−1^ chloramphenicol and incubated at 37°C and 180 rpm. Cells were harvested by centrifugation after 15 to 17 h, resuspended in 100 mM Tris/HCl buffer (pH 8) containing 150 mM NaCl and lysed by sonication. After removal of insoluble cell material by centrifugation (10000 ***g*** for 25 min at 4°C), TsdA or its variants were purified by Strep-Tactin affinity chromatography with a 5 ml StrepTrap HP column (GE Healthcare) according to manufacturer's instructions. Afterwards, a gel filtration chromatography was performed on a HiLoad 16/60 Superdex 75 pg column (GE Healthcare) using an ÄKTApurifier system (GE Healthcare). The column was equilibrated with 20 mM BisTris/HCl buffer, pH 6.5. The purified protein was concentrated with Amicon Ultra-15 30K centrifugal filter units (Merck Millipore) and desalted with a 5 ml HiTrap Desalting column (GE Healthcare). The protein was stored in 50 mM BisTris buffer, pH 6.5 at −70°C. The concentration of purified protein was determined with the BCA Kit from Pierce. For assessment of purity, SDS/PAGE was performed. Haem staining in acrylamide gels followed an established procedure [21]. Haem content was determined by the pyridine haemochrome method [22].

### Construction of *C. jejuni ΔtsdA*/*tsdA complementation strains

The pC46-fdxA-0815HIS complementation vector integrates a copy of the *tsdA* gene at the phenotypically silent *cj0046c* pseudogene locus in *C. jejuni*, with expression driven by the moderately strong *C. jejuni fdxA* promoter. It was constructed by cloning the amplicon resulting from PCR of genomic DNA of *C. jejuni* strain 81116 with primers 0815-com-F/ATG and 0815-com-R5XHis (Table 2) into the BsmBI site of pC46-fdxA (8). The produced TsdA protein has a C-terminal 5 residue His-tag added, that was used for detection in immunoblots (see below). For the TsdA–C138G exchange, two fragments were amplified from pC46-fdxA-0815 with the following primers: for the first fragment pC46_TsdA_Mfe_fw and tsdACj-C-Gly_fw, for the second fragment tsdACj-C-Gly_rev and pC46_TsdA_Cla_rev (Table 2). Both fragments were used as templates for amplification of the complete *tsdA* gene carrying the desired point mutation. In this step, pC46_TsdA_Mfe_fw and pC46_TsdA_Cla_rev served as primers (Table 2). The resulting fragment was restricted with MfeI and ClaI and cloned into pC46-fdxA resulting in plasmid pC46-fdxA-TsdACj-C138G. The following plasmids were generated applying the same general strategy: pC46-fdxA-TsdACj-M255G, pC46-fdxA-TsdACj-N254G and pC46-fdxA-TsdACj-K255G (Table 1). Plasmids were transformed into the *C. jejuni* 81116 Δ*tsdA* mutant strain [2] by electroporation and transformants were selected on Columbia blood agar plates supplemented with chloramphenicol (20 μg·ml^−1^ final concentration). Complementations were confirmed by PCR of genomic DNA with primers pC46F-Scr-F + pC46-Scr-R and Cat-Scr-F + Cj46-Scr-R (Table 2).

### Culture conditions for *C. jejuni* growth

*Campylobacter jejuni* strain 81116 was routinely cultured at 42°C under microaerobic conditions [10% (v/v) O_2_, 5% (v/v) CO_2_ and 85% (v/v) N_2_] in a MACS growth cabinet (Don Whitley Scientific) on Columbia agar containing 5% (v/v) lysed horse blood and 10 μg·ml^−1^ each of amphotericin B and vancomycin. To select *C. jejuni* mutants, kanamycin or chloramphenicol was added at a final concentration of 50 or 20 μg·ml^−1^ respectively. Liquid cultures of *C. jejuni* were routinely grown in Mueller-Hinton broth (Oxoid) supplemented with 20 mM L-serine (MH-S) under standard microaerobic conditions (gas concentrations as above), with 50–100 ml of medium contained in 250 ml conical flasks with continuous orbital shaking at 180 rpm. Except precultures, all cultures contained 50 μM ammonium iron (II) sulfate to induce the *fdxA* promoter in front of *tsdA* in the complementation strains. To study tetrathionate-dependent growth, oxygen-limited cultures were grown in 250 ml of brain heart infusion medium with 20 mM L-serine (BHI-S, brain heart infusion medium supplemented with serine) contained in a 250 ml conical flask with no shaking. In this case, 20 mM sodium formate and 15 mM sodium tetrathionate (final concentrations) were added from filter-sterilized stock solutions. Cultures were maintained in the MACS-VA500 incubator and growth was monitored by measuring absorbance at 600 nm. Values for each growth curve were measured with two independent cultures.

### SDS/PAGE and immunoblotting with *C. jejuni* crude cell extract

*C. jejuni* cells were disrupted by bead beating (Bead Ruptor 12, Omni International) at maximal intensity for 30 s and the crude cell extracts were used for SDS/PAGE. For Western blot analysis, proteins were electroblotted on to nitrocellulose membranes (Amersham Protran 0.45 μm NC, GE Healthcare) for 35 min at 15 V using the Transblot SD semi-dry transfer apparatus (Bio-Rad Laboratories). His-tagged protein was detected with His-tag monoclonal antibody (#70796-3, Novagen) and Goat Anti-Mouse IgG horseradish peroxidase (HRP) Conjugate (#71045-3, Novagen) via the associated peroxidase activity using either chloronaphthol or the SignalFire ECL Reagent kit (Cell Signaling Technology).

### Measurement of tetrathionate reduction and thiosulfate respiration rates

Rates of tetrathionate reduction by cell suspensions were measured by adding washed cells to 14 ml 25 mM phosphate buffer (pH 7.4) containing 20 mM sodium formate. Before addition of cells, the buffer was sparged with N_2_ for 10 min. The buffer-cell suspension was incubated at 42°C for 15 min to allow all of the dissolved oxygen to be consumed. Then, sodium tetrathionate was added to 2 mM final concentration and 0.5 ml samples were taken every 2 or 5 min. The cells were immediately removed from these samples by brief centrifugation (12000 × ***g***, 2 min) and the thiosulfate concentration in the supernatants was measured by the method of Urban [23]. Tetrathionate formation rates were calculated by dividing thiosulfate oxidation rates (μmol·min^−1^·ml^−1^) by two and dividing the result by the protein content. For determination of the protein content, cell suspensions were first incubated at 100°C for 20 min. To remove residual thiosulfate and tetrathionate, which interfere with the BCA assay, an acetone precipitation was performed as the next step: 200 μl of cold (−20°C) acetone was added to 50 μl of the pre-boiled cell suspension, followed by mixing and incubation at -20°C for 1 h. BCA standards were treated accordingly. After centrifugation for 15 min at 15000 × ***g***, the supernatant was discarded. The protein pellets were dried by incubation for 25 min at room temperature and subsequently resuspended in 50 μl of H_2_O. Protein concentration was determined with the BCA Kit from Pierce.

### UV–Vis spectroscopy with TsdA in solution

UV–Vis spectra were recorded with an Analytik Jena Specord 210.

### Assay of tetrathionate reductase activity with reduced methyl viologen

Spectrophotometric measurements of tetrathionate reductase activity were performed with recombinant *C. jejuni* TsdA at 42°C for tetrathionate concentrations up to 0.5 mM and used reduced methyl viologen as electron donor were performed along similar lines as described before [2]. However, in our previous work recombinant enzyme was added to reaction mixtures before these were made anoxic by sparging with argon and addition of 2% titanium (III) citrate oxygen scavenger solution. The assays were started by addition of tetrathionate. Here, enzyme was added to completely oxygen-free reaction mixtures containing tetrathionate and pre-reduced methyl viologen. This improved method yielded 20-fold higher specific tetrathionate reductase activities and thus enabled more precise analysis of kinetic parameters. In addition, the range from 0.01 to 0.5 mM tetrathionate was studied at higher resolution. The assay was carried out in a 1 ml quartz glass cuvette closed with a rubber stopper and purged with N_2_ for 10 min. All buffers and solutions were made anoxic by sparging with N_2_. The final assay mixture contained 100 mM ammonium acetate buffer, pH 5, 0.3 mM methyl viologen reduced with 1–3 μl of 2% titanium (III) citrate oxygen scavenger solution [24] and different final concentrations of tetrathionate. Enzyme solutions were injected after a relatively stable absorbance at 585 nm was achieved (below 2.0). The rate before addition of enzyme was subtracted from the rate after enzyme addition. Control experiments ensured that reduced methyl viologen was provided at a saturating concentration during all measurements with *Cj*TsdA wt and its derivatives. The specific activity for tetrathionate reductase was calculated using molar absorption coefficient for methyl viologen of 11.8 mM^−1^·cm^−1^ at 585 nm. For enzyme activity measurements with crude cell extracts, *C. jejuni* 81116 (wt), Δ*tsdA* mutant (Δ*tsdA*) and the different complementation strains (WT, CG, MG, NG, KG) were first disrupted by bead beating (Bead Ruptor 12, Omni International) at maximal intensity for 30 s. Tetrathionate reduction was measured with 0.1 mM tetrathionate. Protein determination was performed with BCA-Kit from Pierce. Activity is expressed as μmol tetrathionate reduced min^−1^·mg protein^−1^ on the basis of a 1:2 molar ratio of tetrathionate reduced to methyl viologen oxidized.

The activity of TsdA and its variants is inhibited as substrate concentration increases. The *K*_i_ values given in Table 4 were derived from fitting complete data sets to the general equation for substrate inhibition (eqn 1) [25] using Graph Pad Prism (version 6; Graph Pad).

1 $$v\;=\;\frac{V_{\max}\;\left[S\right]}{K_{m\;}+\;\left[S\right]+\;\frac{{\left[S\right]}^{2}}{K_{i}}}$$

Kinetic constants other than *K*_i_ can be derived from eqn (1). In that case, *V*_max_ is the maximum enzyme velocity if the substrate did not inhibit enzyme activity.

### Assay of thiosulfate dehydrogenase activity with horse heart cytochrome *c*

In the article by Liu et al. [2], ferricyanide was used as the artificial electron acceptor during measurements of thiosulfate dehydrogenase activity at pH 4.0. However, a preliminary set of experiments already showed a much lower *S*_0.5_ value for thiosulfate and a near neutral pH optimum of the reaction when horse heart cytochrome *c* was used as electron acceptor [2]. Therefore, all measurements of thiosulfate dehydrogenase activity were performed with horse heart cytochrome *c* (80 μM; C7752, Sigma–Aldrich) as the electron acceptor at 42°C in 50 mM BisTris buffer, pH 6.5. Thiosulfate was varied in the range of 0.05–8 mM. Reactions were started by addition of the enzyme and followed by the increase in absorbance at 550 nm. As the reduction of horse heart cytochrome *c* was measured, a molar absorption coefficient at 550 nm of 21.1 mM^−1^·cm^−1^ [26] was used. The presence of oxygen did not affect these measurements. This was ensured by control reactions with *Cj*TsdA wt and derivatives under anoxic conditions.

In the case of enzymes that use two molecules of the same substrate (here thiosulfate) primary *v* compared with [*S*] plots provide the best way to examine the data [27]. Data were fitted to the empirical Hill eqn (2) using Graph Pad Prism (version 6; Graph Pad).

2 $$v\;=\;\frac{\;V_{\max}{\left[S\right]}^{n}}{K+\;{\left[S\right]}^{n}}$$

The Hill equation resembles the classical Henri–Michaelis–Menten equation; however, the *n* term allows accounting for non-hyperbolic shapes. A substrate concentration [*S*]_0.5_ can be reported that yields half maximal velocity and is characteristic of the process. The constant *K*, which is not equivalent to *K*_m_, characterizes enzyme–substrate interaction. The relationship between *K* and [*S*]_0.5_ is *K*=[*S*]_0.5_*^n^*.

For enzyme activity measurements with crude cell extracts, *C. jejuni* 81116 cells (wild-type), Δ*tsdA* mutant (Δ*tsdA*) and the different complementation strains (WT, CG, MG, NG, KG) were first disrupted by bead beating (Bead Ruptor 12, Omni International) at maximal intensity for 30 s. Thiosulfate oxidation was measured with 2 mM thiosulfate. Protein determination was performed with BCA Kit from Pierce. Activity is expressed as μmol tetrathionate produced per min and mg protein on the basis of one molecule tetrathionate formed per two molecules horse heart cytochrome *c* reduced.

### Protein film cyclic voltammetry of *Cj*TsdA wt, N254G and K252G variant

Experiments were performed with a graphite working electrode as described in [1]. Cyclic voltammograms were measured under identical conditions with the freshly polished electrode placed directly into the desired solution, or, coated with enzyme prior to placement in the desired solution. The catalytic currents (*i*_cat_) due to the activity of the enzyme were defined by subtraction of the response of the bare electrode from that of the enzyme coated electrode. The dependence of *i*_cat_ on potential was independent of whether the scan was to more negative, or more positive, potentials.

### Statistical analysis

Average values and standard deviations for *V*_max_ values shown in Tables 3 and 4 as well as for all values shown in Table 5 were calculated from the appropriate data sets using Microsoft Excel 2010. Standard deviations for all further kinetic parameters shown in Tables 3 and 4 were calculated with Graph Pad Prism (version 6; Graph Pad).

**Table 3:** Tetrathionate reduction catalysed by *Cj*TsdA wt and derivatives Enzyme activity was measured under anoxic conditions at 42°C in 100 mM ammonium acetate buffer (pH 5) with a saturating concentration of 0.3 mM methyl viologen previously reduced with titanium (III) citrate. Tetrathionate was varied between 0.01 to 0.7 mM. The units for *V*_max_ are μmol·min^−1^·mg·protein^−1^. Data sets were fitted to the general equation for substrate inhibition [25]. Ligand changes affected either Haem 1 or Haem 2 as indicated.

	TsdA	*V_max_*_measured_ (units/mg)	*V*_max_ (units/mg)	*K*_m_ (μM)	*K*_i_ (μM)
	WT	1316±38	2328±273	74±14	420±100
	C138G	2.63±0.08	3.7±0.5	34±9	810±440
Haem 1	C138H	0.49±0.01	0.5±0.1	24±10	1150±890
	C138M	0.08±0.01	–	–	–
	M255G	35±1	88±13	317±61	570±160
	N254G	1502±136	3284±457	77±15	220±70
	N254K	216±1	2214±918	360±162	20±10
Haem 2	K252G	2045±112	3823±528	69±15	390±130
	NMG	178±3	412±127	113±51	250±130
	KMG	281±1	652±251	151±84	440±300
	KNMG	95±3	129±16	55±14	1620±970

**Table 4:** Thiosulfate oxidation catalysed by *Cj*TsdA wt and derivatives Enzyme assays were performed at 42°C in 50 mM BisTris buffer (pH 6.5) with a saturating concentration of 80 μM horse heart cytochrome *c* as electron acceptor and 0.05–8 mM thiosulfate. The units for *V*_max_ are μmol·min^−1^·mg·protein^−1^. *v* compared with [*S*] plots were fitted to the Hill equation. Ligand changes affected either Haem 1 or Haem 2 as indicated; *n*, Hill coefficient.

	TsdA	*V*_max_ (units/mg)	*S*_0.5_ (μM)	*n*
	WT	1265±70	280±45	0.87±0.07
	C138G	2.1±0.3	527±273	0.71±0.12
Haem 1	C138H	0.6±0.1	343±205	0.77±0.32
	C138M	0.04±0.01	660±532	0.52±0.13
	M255G	0.7±0.0	162±15	1.07±0.11
	N254G	469±29	194±34	0.94±0.10
	N254K	211±16	157±34	0.95±0.18
Haem 2	K252G	2782±275	330±79	1.24±0.31
	NMG	0.6±0.1	72±27	0.61±0.13
	KMG	0.8±0.1	67±41	0.60±0.18
	KNMG	0.1±0.0	101±29	0.78±0.16

**Table 5:** Growth rate and doubling time of different *C. jejuni* cultures Growth parameters were calculated from *A*_600_ values taken in the exponential phase of the 81116 wt (81116), Δ*tsdA* mutant (Δ*tsdA*) and the different ΔtsdA/*tsdA complementation strains (*tsdA WT, *tsdA C138G, *tsdA M255G, *tsdA N254G, *tsdA K252G). Cells were grown under oxygen-limited conditions at 42°C in almost completely filled 500 ml shake flasks containing BHI-S medium plus 20 mM sodium formate and 15 mM tetrathionate.

Strain	81116	Δ*tsdA*	*tsdA WT	*tsdA C138G	*tsdA M255G	*tsdA N254G	*tsdA K252G
μ	0.12±0.01	0.01±0.00	0.24±0.01	0.01±0.00	0.05±0.00	0.25±0.02	0.19±0.02
*t*_d_ (h)	5.6±0.4	57.8±14.9	2.9±0.2	52.3±11.1	13.4±1.1	2.8±0.3	3.6±0.3

## RESULTS

### Production and characterization of wild-type and variant *Cj*TsdA enzymes

*Cj*TsdA wt and variant proteins (see ‘Materials and Methods’ section for details of construction) were produced recombinantly in *E. coli* BL21 (DE3) containing pEC86 to promote cytochrome *c* synthesis, purified to homogeneity by Strep-tag affinity chromatography and gel filtration. All *Cj*TsdA proteins behaved as homodimers upon analytical gel permeation chromatography (70–76 kDa) and showed the expected size of 37 kDa (exactly: 37103 Da not including the signal peptide, with two covalently bound haems) upon SDS/PAGE (Supplementary Figure S1). Quantification of *Cj*TsdA by the pyridine haemochrome method established that full haem loading had occurred in all cases (1.8–2.2 mol haem per mol protein). Amino acid numbering applies to recombinant protein without signal peptide but including the N-terminal Strep-tag.

### Haem ligation in wild-type *Cj*TsdA

**Figure 2:**
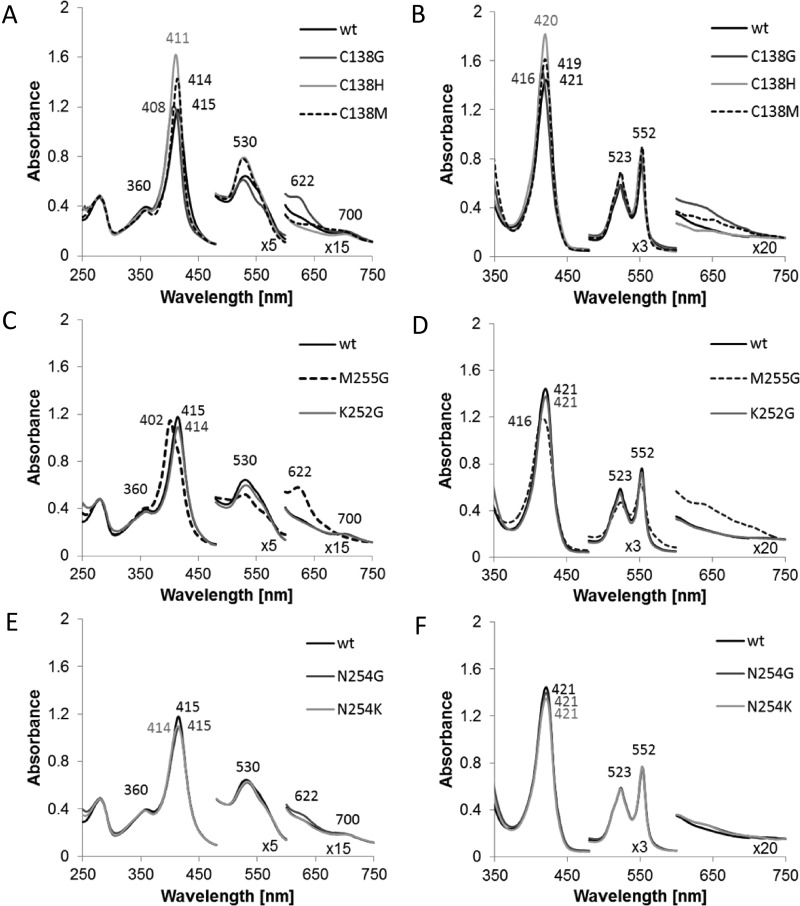
UV–Vis spectra of *Cj*TsdA wt and variants UV–Vis spectra of *Cj*TsdA wt are compared with spectra of variants with affected Haem 1 ligation (**A** and **B**) and with spectra of variants with affected Haem 2 ligation (**C**–**F**). As some of the proteins are partly reduced in the ‘as isolated’ state, up to 30 μM ferricyanide was added to record the oxidized spectrum (A, C and E). For full reduction of the proteins, 5–16 mM Na-dithionite was added (B, D and F). Thirty millimolars BisTris buffer (pH 6.5) were used and spectra were normalized to 280 nm and 750 nm. For *Cj*TsdA M255G and *Cj*TsdA C138G, a high-spin feature appears at 622 nm in the oxidized state. The oxidized spectrum of TsdA wt and all variants except of M255G exhibits a 700 nm peak indicating methionine as haem iron ligand. Protein concentration: 8 μM.

On the basis of amino acid sequence comparisons [2,15], the iron atom of *Cj*TsdA Haem 1 is predicted to be in a six-coordinated low spin state. Strictly conserved histidine (His^99^) and cysteine (Cys^138^) residues are likely to serve as axial haem iron ligands (Figure 1). Accordingly, a replacement of *Cj*TsdA Cys^138^ by a glycine, an amino acid not capable of haem iron co-ordination, led to a low-intensity spectral feature at 622 nm in the oxidized state indicative of the presence of high-spin haem either with water as a sixth ligand or with five-coordinate iron (Figure 2) [28,29] thus confirming Cys^138^ as the sixth haem ligand of Haem 1.

In *Av*TsdA, Haem 2 exhibits axial His/Lys co-ordination in the oxidized state. A ligand switch from the iron-coordinating Lys^208^ to Met^209^ is observed upon reduction of the enzyme [16]. Although the methionine residue is strictly conserved in all TsdA sequences (residue 255 in *Cj*TsdA), variation is apparent at the position of the lysine, which is located one residue closer to the N-terminus (position 254 in *Cj*TsdA). In *Cj*TsdA, an asparagine (Asn^254^) is found instead of the lysine in the *A. vinosum* protein (Figure 1). UV–Vis spectroscopy and amino acid replacement clearly revealed that Met^255^ acts as the sixth axial ligand of Haem 2 iron in *Cj*TsdA, at least in the oxidized state. First, a peak at 700 nm, which is characteristic for methionine as haem iron ligand [28], is clearly apparent in the oxidized spectra of *Cj*TsdA wt (Figure 2A) as well as in *Cj*TsdA variants with N254G, K252G and N254K exchanges (Figures 2C and 2E) but no longer present in the spectrum of a variant with a M255G replacement (Figure 2C). Second, the exchange of Met^255^ to the ligation-incompetent glycine leads to appearance of a high-spin haem feature at 622 nm [28,29] in the oxidized spectrum (Figure 2C).

### Oxidative and reductive assays–experimental approaches and data analysis procedures

**Figure 3:**
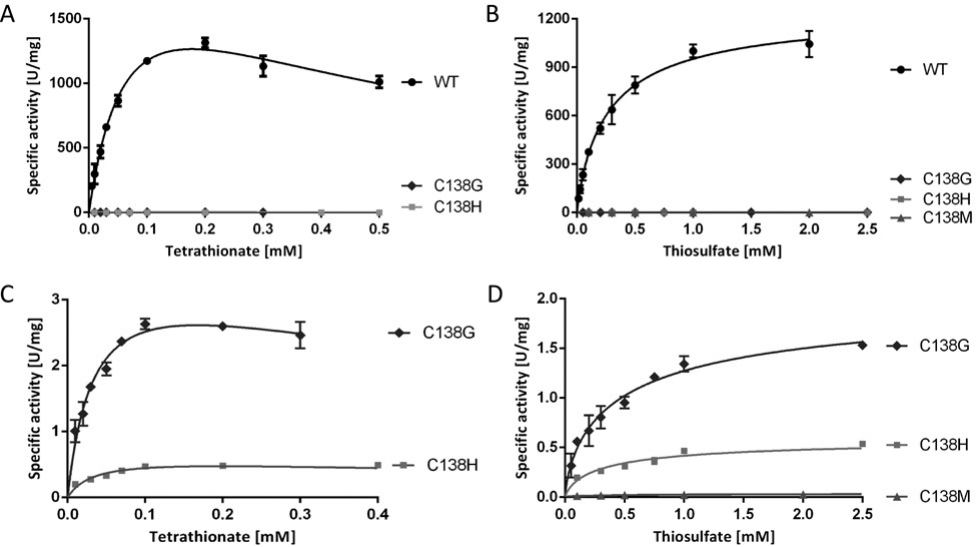
Enzyme activity assays with TsdA wt and Haem 1 ligation affected variants TsdA wt was compared with TsdA C138G, C138H and C138M concerning tetrathionate reduction (**A**) and thiosulfate oxidation (**B**). Tetrathionate reduction (**C**) and thiosulfate oxidation (**D**) of the TsdA C^138^ variants are shown in detail in the panels below. Tetrathionate reduction (A and C) was measured under anoxic conditions at 42°C in 100 mM ammonium acetate buffer (pH 5) with 0.3 mM methyl viologen previously reduced with titanium (III) citrate. Control measurements showed that methyl viologen was provided at a saturating concentration in all cases. Tetrathionate reduction activity of TsdA C138M was so low that exact kinetic parameters could not be derived with confidence. Different tetrathionate concentrations (0.01–0.7 mM) were used. Thiosulfate oxidation (B and D) was measured at 42°C in 50 mM BisTris buffer (pH 6.5) with 80 μM horse heart cytochrome *c* as electron acceptor and 0.05–8 mM thiosulfate. Control measurements showed that horse heart cytochrome *c* was provided at a saturating concentration in all cases.

**Figure 4:**
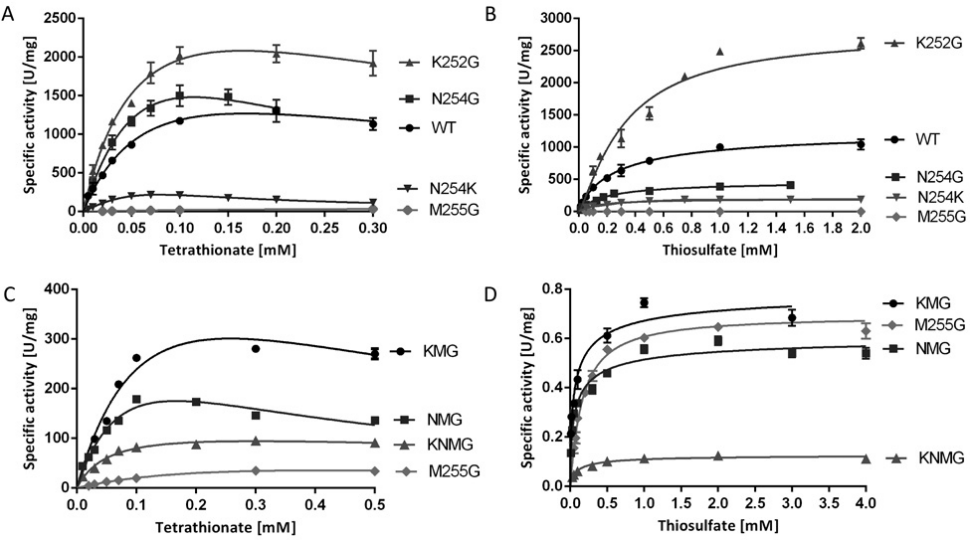
Enzyme activity assays with TsdA wt and Haem 2 ligation affected variants Tetrathionate reduction (**A** and **C**) was measured under anoxic conditions at 42°C in 100 mM ammonium acetate buffer (pH 5) with 0.3 mM methyl viologen previously reduced with titanium (III) citrate. Different tetrathionate concentrations (0.01–0.7 mM) were used. Thiosulfate oxidation (**B** and **D**) was measured at 42°C in 50 mM BisTris buffer (pH 6.5) with 80 μM horse heart cytochrome *c* as electron acceptor and 0.05–8 mM thiosulfate.

Solution assays with redox dyes yielded the first kinetic parameters for *Cj*TsdA [2]. In the present study, we expanded this work to TsdA variants carrying amino acid replacements in the immediate environment of Haem 1 or Haem 2 (Figures 3 and 4, Tables 3 and 4). In addition, parameters for the wild-type enzyme were reassessed under optimized conditions (cf. Materials and Methods). Before describing specific results, some justification of the choice of assays and methods of data analysis are warranted.

*In vivo*, one or more of a set of four monohaems cytochrome *c*’s probably serve as the periplasmic electron carrier mediating electron flow between the cytochrome *bc*_1_ complex and the periplasmic TsdA enzyme [2]. This is substantiated by the finding that neither the dihaem cytochrome *c* TsdB, which functions as natural electron acceptor for TsdA in a number of other organisms [15,30] nor the endogenous *Campylobacter* dihaem cytochrome *c* C8j_0040 (Cj0037 or CccC in the reference strain NCTC 11168 [17] is reduced by *Cj*TsdA *in vitro* [2]. As a consequence, all measurements of thiosulfate dehydrogenase activity reported in the present study were performed at pH 6.5 in the presence of 80 μM horse heart cytochrome *c*. In the direction of thiosulfate oxidation, data sets need to be fitted to the Hill equation [27]. Conventional Michaelis–Menten kinetics does not apply because the enzyme catalyses the reaction of two molecules of the same substrate (here thiosulfate). The Hill equation does not yield *K*_m_ but a substrate concentration [*S*]_0.5_ can be reported that yields half maximal velocity and is characteristic of the process. The wt enzyme yielded a *V*_max_ of 1265±70 U·mg^−1^ and [*S*]_0.5_ of 280±45 μM_._

The assay for tetrathionate reduction used reduced methyl viologen as electron donor. This enzyme activity is inhibited by tetrathionate as a substrate when its concentration is increased (Figure 3). Therefore, data sets have to be fitted to the general equation for substrate inhibition [25], yielding *K*_i_. Kinetic constants other than *K*_i_ can also be derived and are tabulated in Table 3. However, in this case *V*_max_ represents the maximum enzyme velocity and *K*_m_ represents the Michaelis–Menten constant only if the substrate did not inhibit enzyme activity. As such, the calculated *V*_max_ does not yield very useful information for the comparison of different enzyme variants. To circumvent this problem, the highest specific activities actually measured *in vitro* (*V*_max measured_) for *Cj*TsdA and its derivatives are also given in Table 3. The wt enzyme yielded a *V*_max measured_ of 1316±38 U·mg^−1^. Half maximal apparent reaction velocity was reached at 30 μM tetrathionate, whereas the calculated *K*_m_ (disregarding substrate inhibition) was 74±14 μM.

In view of the above considerations, values for the specificity constant *K*_cat_/*K*_m_ cannot be derived for *Cj*TsdA in a meaningful way for either of the two reaction directions. Furthermore, general use of *K*_cat_/*K*_m_ as a comparative index for the catalytic effectiveness of enzyme variants has been seriously questioned [31].

### Identification of Haem 1 as the active site of *Cj*TsdA

In addition to glycine, the Haem 1 iron-ligating Cys^138^ of *Cj*TsdA was replaced by histidine and methionine (Figure 1). Electronic absorption spectra strongly indicated that histidine and methionine acted as haem ligands in the *Cj*TsdA C138H and *Cj*TsdA C138M variants respectively. The Soret peaks increased in intensity in the oxidized as well as in the reduced state (Figures 2A and 2B) consistent with replacement of Cys^138^ with the stronger field ligands histidine or methionine [32] and in contrast with the C138G variant a high-spin haem feature was not observed. Exchange of Cys^138^ by glycine, histidine or methionine led to drastic decrease in enzyme activity in all three cases. Tetrathionate reduction and thiosulfate oxidation were equally affected (Figure 3, Tables 3 and 4).

### Effect of Haem 2 environment on catalytic properties

As Met^255^ was clearly identified as an important distal ligand of Haem 2 iron, this residue was changed to glycine. Furthermore, Asn^254^ and Lys^252^ were also replaced by glycine (Figure 1). These amino acids are in close vicinity to Haem 2 iron and have theoretical competence for temporary haem ligation. Exchange of Met^255^ had a dramatic effect on catalysis, with *V*_max_ in the thiosulfate-oxidizing direction reduced by a factor of 1900 as compared with the wild-type protein (Figure 4, Table 4). Thiosulfate oxidation was much more impaired than tetrathionate reduction (Tables 3 and 4, Figure 1). The *Cj*TsdA K252G variant exhibited a higher *V*_max_ than the wild-type enzyme in both catalytic directions with a significantly stronger improvement in the oxidative direction. In contrast, the *Cj*TsdA N254G variant exhibited virtually unaltered catalytic parameters in the tetrathionate-reducing direction, but a much lower activity in the oxidative direction than the wild-type enzyme (Tables 3 and 4). Thus, the N254G variant appears more adapted to catalysing the reductive direction.

In an attempt to create a haem environment in *Cj*TsdA that resembles the situation in *Av*TsdA, Asn^254^ was replaced by lysine. This variant exhibited especially pronounced substrate inhibition by tetrathionate with a *K*_i_ of 0.02 mM as compared with 0.42 mM for the wild-type enzyme (Table 3, Figure 4). As a consequence, the calculated *V*_max_ exceeded the highest measurable specific activity by a factor of ten. *V*_max measured_ for this variant was 6-fold lower than that for *Cj*TsdA wt. Catalysis in the thiosulfate-oxidizing direction was affected in a comparable manner.

When Asn^254^ or Lys^252^ or both residues were changed in addition to Met^255^, resulting in the *Cj*TsdA KNG, NMG or KNMG derivatives, highest measured *V*_max_ increased in the tetrathionate-reducing direction in all three cases when compared with the M255G variant (Table 3, Figure 4C). In contrast, in the thiosulfate-oxidizing direction the activity of the variants carrying double and triple replacements was impaired in a similar manner as observed for the M255G single mutant (Table 4, Figure 4D). This indicates that a lack of Asn^254^ and/or Lys^252^ has a positive effect on tetrathionate reduction. The triple mutant exhibited the lowest specific activity.

**Figure 5:**
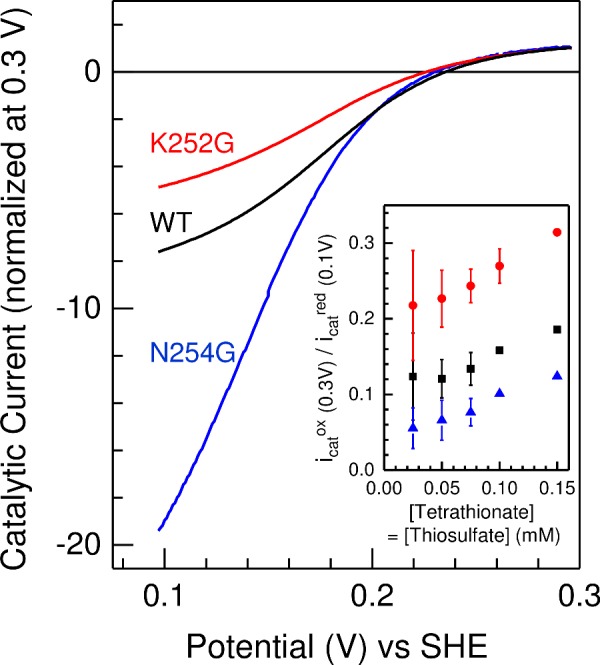
Baseline subtracted protein film cyclic voltammetry of *Cj*TsdA wild-type (black), N254G (blue) and K252G variant (red) in 0.05 mM tetrathionate and 0.075 mM thiosulfate **Inset**: The dependence of *i*_cat_^ox^/*i*_cat_^red^ on substrate concentration for equimolar substrate concentrations; wild-type (black), N254G (blue) and K252G variant (red), see text for details. Values are the average of two independent measurements, errors show maximum and minimum values, except for N254G at 0.1 and 0.15 mM where single values are presented. Scan rate 10 mV·s^−1^, electrode rotation 500 rpm in 100 mM ammonium acetate, 50 mM NaCl, pH 5 at 42°C.

Taken together, the above results indicate that the Haem 2 environment has an impact on the catalytic directionality of *Cj*TsdA. Recently, a catalytic bias towards tetrathionate reduction was demonstrated for *Cj*TsdA by protein film voltammetry in solutions containing equal concentrations of thiosulfate and tetrathionate [1]. In these experiments, the enzyme was adsorbed in an electrocatalytically active form on graphite electrodes such that the electrode serves both an electron-accepting and an electron-donating function depending on the potential. Thus, the same reaction is assayed in the forward and the reverse direction and any differences in rate that may be introduced by using different redox dyes for the two different directions are eliminated. As a consequence, we compared protein film voltammetry of *Cj*TsdA WT with that of the two most active variants with an altered Haem 2 environment, TsdA N254G and K252G, to provide an independent measure of the relative catalytic directionality of these proteins. Cyclic voltammetry was performed in solutions containing tetrathionate and thiosulfate at equal concentration for values between 0.025 mM and 0.15 mM (Figure 5). In each case, the negative catalytic currents (*i*_cat_^red^) that quantify tetrathionate reduction reached greater magnitudes than the positive catalytic currents (*i*_cat_^ox^) that quantify thiosulfate oxidation. To assess catalytic bias, values for *i*_cat_^red^ and *i*_cat_^ox^ were measured at 0.1 and 0.3 V compared with SHE respectively, where there is equal thermodynamic driving force (0.1 V) for each reaction. For a given substrate concentration, *i*_cat_^ox^ (0.3 V)/*i*_cat_^red^ (0.1 V) followed the order K252G > WT > N254G (Figure 5, *inset*), which confirms the catalytic directionality deduced from the spectrophotometric assays (Tables 3 and 4). Because the voltammetric experiments were performed in equimolar concentrations of tetrathionate and thiosulfate, the catalytic rate for each reaction is defined by factors that include not just the *K*_m_ and *V*_max_ values for the relevant substrate but also the impact of substrate and production inhibition on each reaction [1]. As a consequence, comparison of the magnitude of *i*_cat_^ox^ (0.3 V)/*i*_cat_^red^ (0.1V) with a simple counterpart deduced from the spectrophotometric analyses is not warranted.

### Impact of the immediate haem environments on the *in vivo* catalytic properties of *Cj*TsdA

The *in vitro* studies on recombinant *Cj*TsdA and its variants were complemented by analysing the extent to which substitutions of the Haem 1 ligand Cys^138^ and the immediate Haem 2 environment (M255G, N254G and K252G) affect the activity of *Cj*TsdA *in vivo* (i.e with the correct natural physiological electron donor/acceptor) and whether the changes can be correlated to the observations *in vitro.* Selected *Cj*TsdA variants studied above were produced in the original host *C. jejuni* strain 81116 in order to study the influence of alterations in *Cj*TsdA axial haem ligation on growth and tetrathionate reduction. To investigate the role of Haem 1 ligation, we employed the C138G variant. However, as our major focus was on the effect of changing the environment of Haem 2 on *in vivo* activities, we chose the M255G, N254G and K252G variants for particular attention. The previously constructed *tsdA* deletion mutant [2] was complemented by integration of either the wild-type or the *tsdA* genes encoding these variant proteins at the *cj0046* pseudogene locus, using a vector in which gene expression was driven by the strong *fdxA* (ferredoxin) promoter from *C. jejuni*. Strains with complemented *tsdA* genes produced C-terminally His-tagged *Cj*TsdA enabling Western Blot analysis, thus allowing a comparison of the amounts of wild-type and TsdA variants *in vivo*.

**Figure 6:**
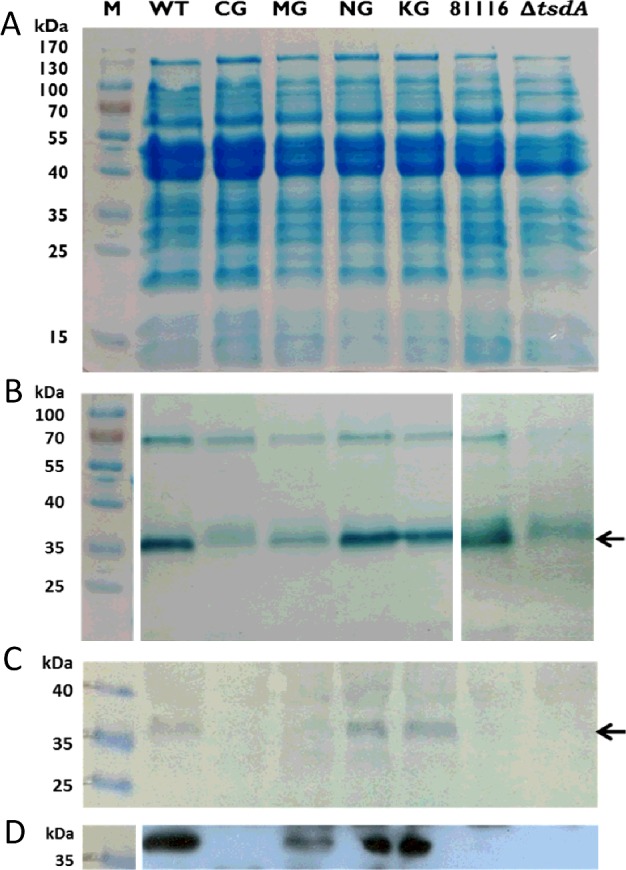
SDS/PAGE of crude cell extracts of *C. jejuni* 81116 wt, Δ*tsdA* mutant and complementation strains *C. jejuni* cells of 81116 wt (81116), Δ*tsdA* mutant (Δ*tsdA*) and the different complementation strains (*tsdA WT, *tsdA C138G, *tsdA M255G, *tsdA N254G, *tsdA K252G) were disrupted by bead beating and the crude cell extracts were used for SDS/PAGE. Forty micrograms of protein per lane were loaded on the gels for Coomassie staining (**A**) and Western blots (**C** and **D**), and 27 μg of protein per lane was used for haem staining (**B**). For the Western blots, antibody against His-tag was used and the second antibody was a HRP conjugate. The detection was based on the reaction of HRP with chloronaphthol (C) or the interaction between HRP and ECL reagent (D). The 37 kDa protein *Cj*TsdA is marked with an arrow. The approximately 70 kDa haem stained band in panel (B) is the MccA multi-haem sulfite reductase (see text).

SDS/PAGE analysis of crude cell extracts of *C. jejuni* 81116, the Δ*tsdA* mutant and the ΔtsdA/*tsdA complementation strains did not reveal a specific stained protein band correlating in size with TsdA (37103 Da; without signal peptide, with haems) (Figure 6A). Under the conditions applied, TsdA thus does not appear to be of major abundance in *C. jejuni.* Haem staining (Figure 6B) as well as Western blot analysis (Figures 6C and 6D) clearly revealed that the *tsdA wt, *tsdA N254G and *tsdA K252G complementation strains produced the different TsdA variants in comparable amounts. TsdA M255G was produced in a lower amount and TsdA C138G was hardly detectable. Either these variants are inefficiently produced or are degraded in *C. jejuni*. *C. jejuni* 81116 as well as the Δ*tsdA* mutant do not produce His-tagged protein and were used as negative controls in Western Blot analysis. The haem stained gel shows that less TsdA is formed in *C. jejuni* 81116 than in the ΔtsdA/*tsdA wt complementation strain, which suggests that the *fdxA* promoter in front of *tsdA* in the complementation strains is stronger than the natural *tsdA* promoter. For the Δ*tsdA* mutant, a protein was detected after haem staining, which is slightly larger than TsdA (Figure 6B). This band is likely to be C8j_0040, the dihaem cytochrome *c* equivalent to CccC in *C. jejuni* NCTC 11168 [17]. C8j_0040 has detectable sequence homology to TsdA, but does not exhibit clear enzyme activity [2]. The uppermost visible band in the haem stained gels can be unequivocally identified as the 71 kDa protein MccA, a multi-haem sulfite reductase, which is the largest *c*-type cytochrome encoded in the strain 81116 genome [17].

**Figure 7:**
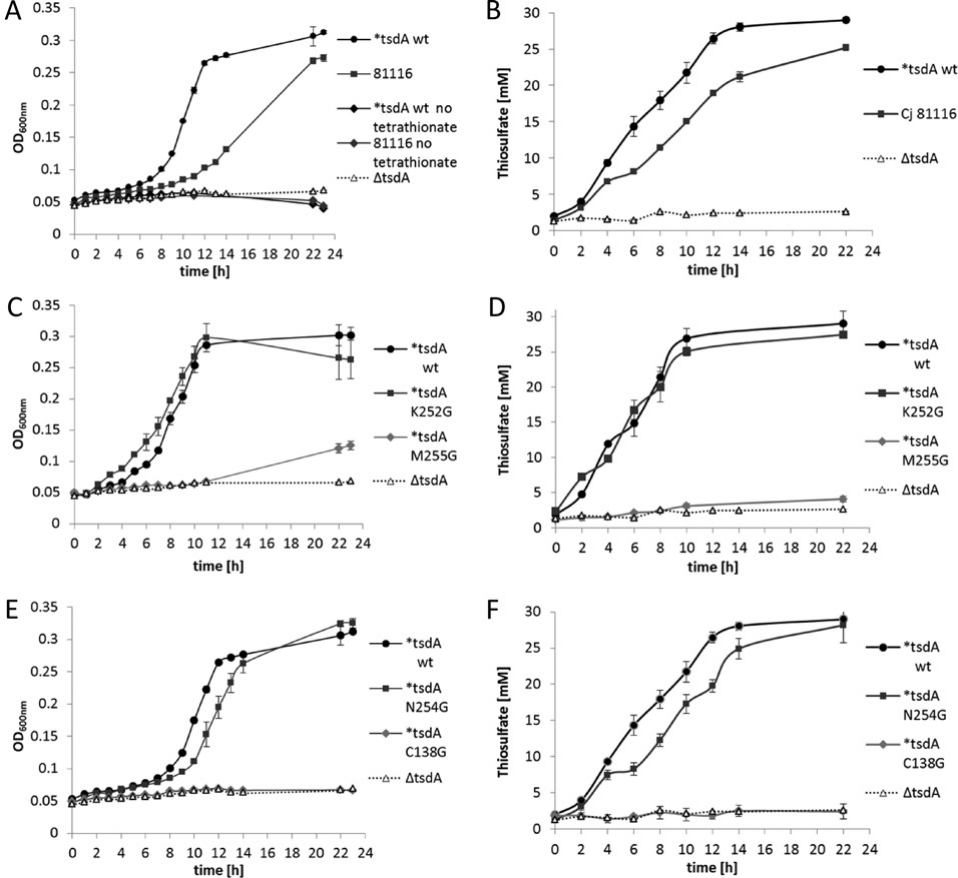
Anaerobic growth of *C. jejuni* 81116 wt, Δ*tsdA* mutant and complementation strains *C. jejuni* cells of 81116 wt (81116), Δ*tsdA* mutant (Δ*tsdA*) and the different ΔtsdA/*tsdA complementation strains (*tsdA WT, *tsdA C138G, *tsdA M255G, *tsdA N254G, *tsdA K252G) were grown under oxygen-limited conditions at 42°C in almost completely filled 500 ml shake flasks containing BHI-S medium plus 20 mM sodium formate and 15 mM tetrathionate. In (**A**, **C** and **E**), the absorbance at 600 nm of the wild-type 81116 strain (closed black circles) is compared with that of an 81116 *tsdA* mutant (open triangles) and the different ΔtsdA/*tsdA complementation strains. In the absence of added tetrathionate [dark grey and black diamonds in (A)], no growth of either strain occurred. In (**B**, **D** and **F**), the conversion of tetrathionate to thiosulfate in each of the culture supernatants at each time point corresponding to the growth curves in (A, C and E) is shown.

Growth experiments were performed with *C. jejuni* 81116, the Δ*tsdA* mutant and the different ΔtsdA/*tsdA complementation strains to analyse the impact of changes in TsdA haem ligation on the growth of *C. jejuni* with tetrathionate as electron acceptor. The cells were grown under oxygen-limited conditions at 42°C in almost completely filled 500 ml shake flasks containing BHI-S medium plus 20 mM sodium formate and 15 mM tetrathionate. As a control experiment, *C. jejuni* 81116 and the ΔtsdA/*tsdA wt complementation strain were grown with and without tetrathionate (Figure 7A). As the strains did not grow without added tetrathionate, but did grow with it, we ensured that the growth of the different *C. jejuni* strains used in this experiment is solely supported by tetrathionate as electron acceptor and not by residual oxygen in the medium. The Δ*tsdA* mutant did not grow with tetrathionate (Figure 7A) confirming that TsdA is indispensable for tetrathionate respiration in *C. jejuni.* The ΔtsdA/*tsdA wt complementation strain grew faster than the wild-type strain and exhibited a 2-fold lower doubling time (Table 5) indicating again that the *fdxA* promoter in front of *tsdA* in the complementation strains is stronger than the natural *tsdA* promoter. In accordance, the ΔtsdA/*tsdA wt complementation strain converted 15 mM tetrathionate much faster into 30 mM thiosulfate than the *C. jejuni* 81116 strain (Figure 7B).

As shown in Figure 6, only the *tsdA wt, *tsdA N254G and *tsdA K252G complementation strains produced the respective TsdA variants in similar amounts and are thus comparable between each other. The ΔtsdA/*tsdA NG and *tsdA KG complementation strains all show growth behaviour (Figures 7C and 7E) and doubling times (Table 5) similar to the ΔtsdA/*tsdA wt complementation strain. Moreover, all these strains converted 15 mM tetrathionate completely into thiosulfate (Figures 7D and 7F). Obviously, growth is not limited by this step of the respiratory chain. The ΔtsdA/*tsdA MG complementation strain showed only very little growth after several hours of incubation (Figure 7C, Table 5) and only small amounts of thiosulfate were produced (Figure 7D). This strain did not produce high amounts of TsdA M255G (Figure 6). The ΔtsdA/*tsdA C138G complementation strain showed a similar growth behaviour as the Δ*tsdA* mutant (Figure 7E, Table 5) and thiosulfate was not formed (Figure 7F).

**Figure 8:**
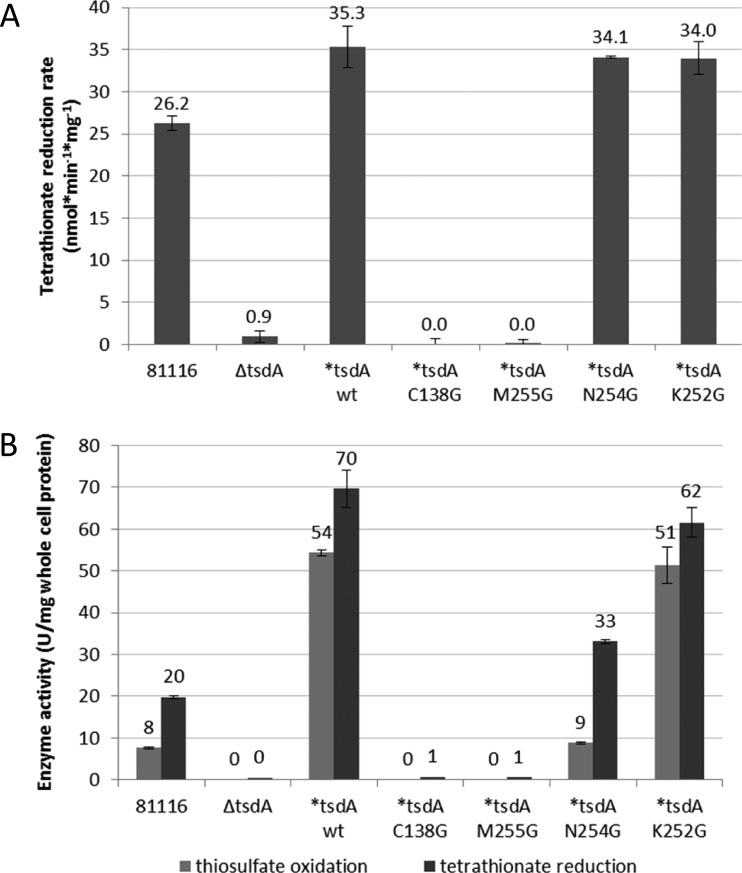
Determination of tetrathionate reduction rate with whole cells and enzyme activity assays with crude cell extract For determination of tetrathionate reduction rate with whole cells (**A**) *C. jejuni* cells of 81116 wt (81116), Δ*tsdA* mutant (Δ*tsdA*) and the different complementation strains (*tsdA WT, *tsdA C138G, *tsdA M255G, *tsdA N254G, *tsdA K252G) were incubated at 42°C in 14 ml phosphate buffer (pH 7.4) containing 20 mM sodium formate and 2 mM tetrathionate. The thiosulfate concentrations in the supernatants of samples taken at 2 or 5 min intervals were determined directly after the experiment. For enzyme activity assays with crude cell extract (**B**) *C. jejuni* cells of 81116 wt (81116), Δ*tsdA* mutant (Δ*tsdA*) and the different complementation strains (*tsdA WT, *tsdA C138G, *tsdA M255G, *tsdA N254G, *tsdA K252G) were disrupted by bead beating. Tetrathionate reduction was measured anaerobically at 42°C in 100 mM ammonium acetate buffer (pH 5) with 0.3 mM methyl viologen previously reduced with titanium (III) citrate and 0.1 mM tetrathionate. Thiosulfate oxidation was performed at 42°C in 50 mM BisTris buffer (pH 6.5) with 80 μM horse heart cytochrome *c* as electron acceptor and 2 mM thiosulfate.

In order to evaluate the rate of electron transfer to TsdA *in vivo*, the formate-dependent tetrathionate reduction rate over a short time period was determined with resting cells of *C. jejuni* 81116, the Δ*tsdA* mutant and the different ΔtsdA/*tsdA complementation strains (Figure 8A). The ΔtsdA/*tsdA wt, the *tsdA K252G and the *tsdA N254G complementation strains did not show large differences in the tetrathionate reduction rate. The *C. jejuni* 81116 strain exhibits a lower tetrathionate reduction rate than the ΔtsdA/*tsdA wt complementation strain, because this strain does not produce as much TsdA as the complementation strains (Figure 8). The ΔtsdA/*tsdA M255G and the *tsdA C138G complementation strains as well as the Δ*tsdA* mutant do not reduce tetrathionate, as previously discussed.

In a last set of *in vivo* experiments, we used crude cell extracts of the same strains to measure specific enzyme activities (Figure 8B). *C. jejuni* strain 81116 showed much higher tetrathionate reduction than thiosulfate oxidation activity, demonstrating that wild-type *Cj*TsdA works best as a tetrathionate reductase and agreeing with earlier results on the pure enzyme [2]. The activities of the ΔtsdA/*tsdA wt complementation strain are higher than those of *C. jejuni* 81116 in both directions, as more TsdA is produced in this strain. The Δ*tsdA* mutant, the ΔtsdA/*tsdA M255G and the *tsdA C138G complementation strains did not show enzyme activity (Figure 8B), explaining the inability of the respective strains to reduce or to grow on tetrathionate. The N254G and K252G complementation strains showed tetrathionate reductase activity of same order of magnitude as for WT (consistent with expression levels and growth phenotypes).

## DISCUSSION

In the present study, we assessed how the nature of the haem environments alters the catalytic properties of the dihaem cytochrome *c* tetrathionate reductase/thiosulfate dehydrogenase TsdA *in vivo* and *in vitro* and chose the enzyme from *C. jejuni* as an example. First, we demonstrated without ambiguity that Haem 1 in *Cj*TsdA is axially ligated by a cysteine (Figure 1). Haem 1 constitutes the active site of *Cj*TsdA, as enzyme activity is almost completely abolished in both directions when the Haem 1 ligating Cys^138^ is replaced. Only very recently it has been unambiguously demonstrated that catalysis of thiosulfate oxidation by TsdA and very probably also that by the other *c*-type cytochrome catalysing an oxidative reaction affecting thiosulfate, namely SoxXA, involves formation of a covalent adduct between the sulfane sulfur atom of thiosulfate and the S_γ_ of the active site cysteine [30]. The finding that the *Cj*TsdA C138G, C138M and C138H variants still exhibit some residual activity shows that the covalent reaction intermediate is not absolutely required for catalysis. In *Cj*TsdA, a change of Cys^138^ to iron-ligation competent histidine or methionine impaired catalysis much more than a replacement by glycine (Figure 1, Table 1), which cannot ligate the haem iron and yields a significant enzyme population containing five-coordinated high-spin haem or high-spin haem with water as a sixth ligand (Figure 2A). This shows that the temporary presence of five-coordinated high-spin haem, which cannot be generated in the C138H and C138M variants, is an important though not absolutely essential prerequisite for the reaction to proceed. Thus, the concept is strengthened that movement of the Haem 1 ligating cysteine out of the haem iron co-ordination sphere and covalent attachment of thiosulfate to the cysteine's S_γ_ in this state is a central part of the TsdA reaction cycle [16,33]. The finding that a cysteine S-thiosulfonate intermediate is formed during thiosulfate oxidation catalysed by TsdA in combination with the reaction mechanism proposed by Grabarczyk et al. [34] for *Av*TsdA catalysis led to the conclusion that in case of thiosulfate oxidation formation of the cysteine S-thiosulfonate releases two electrons that reduce the iron atoms of the two haems in TsdA to the Fe(II) state. After haem reoxidation by an external electron acceptor a thiol–disulfide exchange reaction is likely to proceed via an attack of the sulfane atom of a second thiosulfate molecule on the thiosulfonate group. In the tetrathionate-reducing direction, the central sulfur–sulfur bond of the tetrathionate molecule first would be cleaved by attack of the active site cysteine's S_γ_ atom thus releasing the first thiosulfate molecule and creating the cysteine S-thiosulfonate. From this intermediate, the second thiosulfate would be reductively released with two electrons delivered by an external electron donor via the two TsdA haem groups.

TsdA Haem 2 acts as an electron relay centre and wires the active site to the enzyme's redox partner [16]. An important difference between the tetrathionate-reduction adapted *Cj*TsdA and the thiosulfate-oxidizing enzyme from *A. vinosum*, is indeed apparent at this haem. At *Av*TsdA Haem 2, a ligand change occurs from lysine to methionine upon reduction [16]. In contrast, we found for *Cj*TsdA that the equivalent methionine residue is ligating Haem 2 iron in the oxidized state. The replacement of Met^255^ by glycine led to a strong impairment in both catalytic directions, however the effect on tetrathionate formation was much more severe. The simple attempt to create a haem environment in *Cj*TsdA resembling the situation in *Av*TsdA by exchanging Asn^254^ for lysine did not have the expected effect. As evidenced by UV–Vis spectroscopy, the exchange did not lead to a replacement of Met^255^ as the haem iron ligand by the newly introduced lysine in the oxidized state (Figure 2E). Furthermore, the replacement negatively affected *V*_max_ in both the tetrathionate-forming and the tetrathionate-reducing direction and did not cause adaptation of TsdA to catalysing thiosulfate oxidation as seen for *Av*TsdA.

Unexpectedly, the largest changes in *Cj*TsdA reaction directionality and catalytic efficiency were observed upon replacing Asn^254^ by glycine instead of lysine and exchanging Lys^252^ to glycine (Figures 1 and 5) demonstrating the importance of those amino acid residues for functionality of the enzyme. The K252G exchange improved *Cj*TsdA, resulting in higher *V*_max_ in both catalytic directions. However, the reaction directionality of this variant also appeared to shift towards thiosulfate oxidation in solution assays and this was substantiated by protein film electrochemistry. In contrast, the *Cj*TsdA N254G variant exhibited lower specific activity in the oxidative direction than the wild-type enzyme and proved more adapted to catalysing the tetrathionate-reducing direction in electrochemical experiments. These differences might be explicable by altered redox potentials of Haem 2, but more subtle changes in intramolecular electron transfer or reorganization energy might also apply [35,36].

A surprising level of complexity became apparent through the finding that replacement of Asn^254^ by glycine or lysine leads to a much stronger substrate inhibition by tetrathionate than observed for wild-type *Cj*TsdA. This demonstrates that alterations at Haem 2 have an effect on the active site haem and its catalytic properties, indicating interaction between the two haem groups of TsdA. This is corroborated by the finding that the *K*_m_ and *S*_0.5_ values vary between the *Cj*TsdA derivatives with altered Haem 2 ligands (Tables 3 and 4). The same has been observed for *Av*TsdA [16].

Adaptation of a given TsdA to catalyse one or the other reaction direction, i.e. tetrathionate reduction or thiosulfate oxidation should have implications on its function in metabolic networks *in vivo*, i.e. how effectively tetrathionate reduction can be used as an alternative respiratory process. In the present study, we assessed this question by complementing a *C. jejuni* 81116 mutant strain devoid of the *tsdA* gene with TsdA variants exhibiting different specific activity and catalytic adaptation. The C138G variant lacking the active site cysteine was produced in very small amounts (Figure 6) and its specific activity was very low. It was therefore not surprising that this complementation strain was unable to grow with tetrathionate as electron acceptor. The same holds true for the M255G variant, which did not support significant growth with tetrathionate as respiratory electron acceptor. The catalytic properties of TsdA measured in crude cell extracts of the ΔtsdA/*tsdA wt complementation strain appear similar to those of pure recombinant TsdA wt (Tables 3 and 4, Figure 8B). Crude extracts of the ΔtsdA/*tsdA K252G complementation strain showed activities very similar to the ΔtsdA/*tsdA wt complementation strain. For the ΔtsdA/*tsdA N254G complementation strain, enzyme activities per mg cell protein were not as high as for the ΔtsdA/*tsdA wt complementation strain, but activity in the thiosulfate oxidizing direction was much more negatively affected than tetrathionate reduction. Growth characteristics for the strains complemented with the wild-type enzyme and the *Cj*TsdA N254G and K252G variants did not differ significantly. This was surprising for the *tsdA K252G complementation strain because the TsdA K252G variant clearly exhibited higher specific activity than the wild-type enzyme *in vitro* and was present in similar amounts (Figure 6). We conclude that tetrathionate reduction by TsdA is not the limiting step in the respiratory chain of *C. jejuni* under the conditions applied.

In conclusion, we identified the haem iron ligands of *Cj*TsdA and showed that Haem 2 iron ligation is different to that observed for *Av*TsdA in the oxidized state. The Haem 1 axial ligand cysteine and the Haem 2 iron-ligating methionine are indispensable for efficient enzyme function *in vitro* and *in vivo*. Structural differences in the immediate environment of Haem 2 were shown to contribute to defining the reaction directionality. Ongoing studies in our laboratories aim to gain further understanding of the factors that define the catalytic bias of TsdA enzymes.

## Abbreviations

BHI-S: brain heart infusion medium supplemented with serine
HRP: horseradish peroxidase
SHE: standard hydrogen electrode
TsdA: thiosulfate dehydrogenases/tetrathionate reductases
wt: wild-type
